# Nutrition, a health technology that deserves increasing interest among HTA doers. A systematic review

**DOI:** 10.3389/fphar.2015.00156

**Published:** 2015-07-29

**Authors:** Iñaki Gutiérrez-Ibarluzea, Eunate Arana-Arri

**Affiliations:** ^1^Osteba, Basque Office for Health Technology Assessment, Ministry for HealthVitoria-Gasteiz, Spain; ^2^Clinical Epidemiology Unit, Cruces University Hospital, Osakidetza, Basque Health ServiceBarakaldo, Spain

**Keywords:** nutrition, Health Technology Assessment, health technology, decision making, public health interventions

## Abstract

The increasing interest for evaluating indirect consequences of health care interventions and their interaction with patients' behavior have put the focus on health promotion interventions including nutrition and the need to measure and evaluate them.

**Objective:** In this review we have aimed to analyze current status of written and published reports on nutrition and nutrition interventions by HTA doers, how assessment has been approached and which metrics and designs have been proposed.

**Methods:** For that purpose, we searched the Center for Reviews and Dissemination databases (CRD) comprising the International Network of Agencies for HTA database (INAHTA), the database of effects (DARE) and the National Health Service Economic Evaluation Database (NHS EED). The words used include nutrition and nutrition interventions and there was no limit on data coverage. We complemented the search by manually seek for further reports on INAHTA's agencies webpages. We extracted the reports for their classification and analysis.

**Results:** We found 82 reports from different sources and after applying inclusion and exclusion criteria, we finally included 42. All the reports correspond to High income Countries (HiC) including agencies from Europe, North America and Oceania. The agencies or programs most represented correspond to the NIHR (UK) and AHRQ (USA). There were general reports around the role of functional foods and specific reports on the impact of establishing nutrition specific strategies in hospitals. 6 out of 42 analyzed the economic consequences of nutrition interventions and 4 reports were related to the methodologies used or the appliance of systematic review methods to the field of nutrition.

**Conclusions:** the reports included correspond to HiC while those HTA agencies established in Low and Middle Income countries (LMiC) have no reported or written activities on the role of nutrition and nutrition interventions. Retrieved reports written by HTA doers/producers confirm the use and utility of systematic reviews and economic analysis methods and its applicability for nutrition interventions. However, some measurements such as Quality Adjusted Life Years (QALY) need to be refined to better reflect the impact of these interventions.

Health technology assessment (HTA) is a multidisciplinary field of knowledge that addresses the different consequences of health technologies both direct and indirect to health systems. It embraces the analysis of clinical issues, economic aspects, organizational, legal, social, cultural and ethical issues that one single or a group of technologies can generate. Classically HTA has evaluated drugs, medical devices, surgical procedures and companion diagnostics while public health interventions including vaccines have been sporadically assessed. Nevertheless, and according to the definition of health technology from the HTAi glossary (http://htaglossary.net/HomePage)^1^ it is an intervention that may be used to promote health, to prevent, diagnose or treat acute or chronic disease, or for rehabilitation. It includes pharmaceuticals, devices, procedures and organizational systems used in health care. There is an increasing interest in evaluating public health interventions and its consequences on health and health care systems. This relates not only to the existing initiatives but to the metrics used in evaluating them in order to justify further interventions. In fact, an active group started writing different papers on the role of nutrition and especially its economic impact and the ways of measure nutrition interventions (Lenoir-Wijnkoop et al., 2011, 2012; Gyles et al., 2012). This initiative was further discussed by some members of the international society Health Technology Assessment international (HTAi) which led to the creation of an Interest Subgroup (ISG) in the society. Interest Subgroups are hubs for sharing international experiences and expertise among HTA users and producers worldwide. This newly created ISG on the Impact of Public Health interventions, special focus on Nutrition, on Health Outcomes Research and Measurement (INPHORM) aimed to create a critical mass for discussion and a neutral forum for individuals who are involved in the research, assessment and/or management of public health interventions and a special focus nutrition-related health states and socio-economic outcomes from a broad perspective, including the individual and the societal level. In order to analyze the current situation of HTA analysis in the field of nutrition a systematic overview of existing written reports of the International Network of Agencies for Health Technology Assessment (INAHTA) was proposed. INAHTA is a network of 55 HTA agencies that support health system decision making that affects over 1 billion people in 33 countries around the globe. With more than 2,100 staff and consultants working in the INAHTA network (http://www.inahta.org) and a common database coordinated and organized by the Center for Reviews and Dissemination (CRD) of the University of York, INAHTA is the biggest community of HTA doers/producers. On the other hand, the CRD databases are updated daily and provide decision-makers with access to: over 30,000 quality assessed systematic reviews; over 16,000 economic evaluations and over 13,000 summaries of completed and ongoing health technology assessments. The CRD is the biggest database of HTA reports in the world and includes not only the reports from INAHTA members but from other HTA doers.

## Objective

To conduct a systematic review of the HTA reports that analyzed nutrition and nutrition interventions from the main HTA organizations and doers.

## Methods

We searched the CRD databases comprising the International Network of Agencies for HTA database (INAHTA), the database of effects (DARE) and the National Health Service Economic Evaluation Database (NHS EED). They are updated daily and include more than 60,000 registries of systematic reviews, economic evaluations and HTA reports. The search was complemented with a hand search of the webpages of the agencies members of INAHTA (currently 55 agencies from 33 countries including hospital based HTA units). There was no limit of publication data, the languages that were included were as follows: Spanish, English, Italian, German, French and Portuguese. Inclusion criteria were: HTA reports, economic evaluations or systematic reviews that embrace nutrition and nutrition interventions and that were included in the CRD databases or were available in the INAHTA's agencies webpages. We did not exclude reports if written in other languages if they had a comprehensive abstract with sufficient information in the previously reported languages. The search was closed the 31st of January 2015. Exclusion criteria comprise reports that had at least no abstract in the mentioned languages, which were related to other type of interventions different to nutrition (although they collaterally cited nutrition) or that the abstract was insufficient to provide enough information and the report was written in languages different to the included ones. Inclusion and exclusion criteria were independently applied by both researchers and discrepancies were solved by consensus among them. Finally included reports were classified in three main blocks: methodological studies, studies of effectiveness and reports that include economic analysis. In the case of those studies that report economic evaluation, the perspective used (health system or societal) was analyzed. We also extracted data on publication date, language, HTA agency, country and setting.

## Results

**Figure 1:**
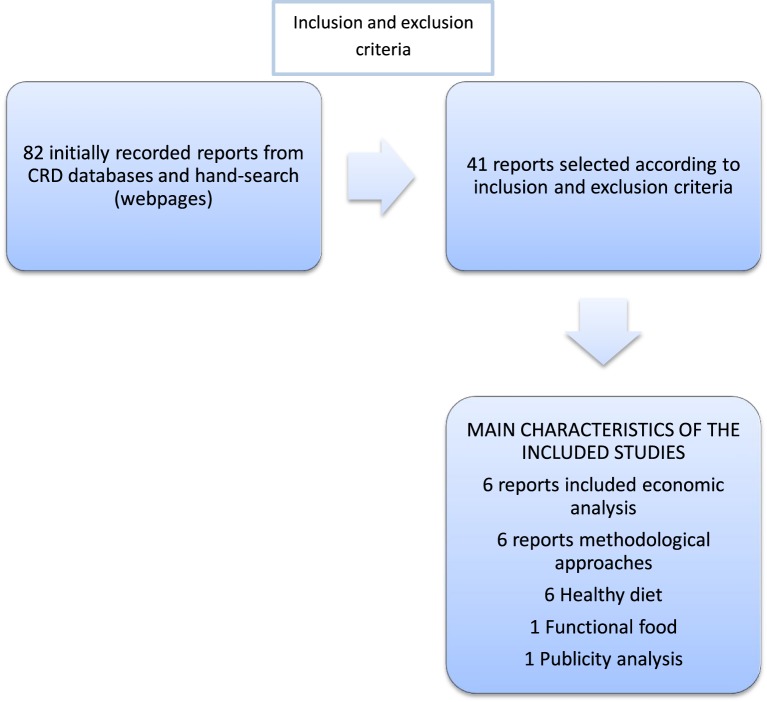
**Diagram of the performed search**.

We retrieved 82 reports (see Figure 1) from different agencies, after the application of inclusion and exclusion criteria, we finally included 42 reports (see Table 1). Fifteen reports were excluded due to the no-existence of comprehensive abstract in the languages considered, 20 due to interventions considered different to nutrition, one report was duplicated (protocol and study results) and 4 were finally excluded due to insufficient information from abstract and impossibility to retrieve further information from the agencies' webpage. The 42 finally considered reports were produced by 16 agencies from 10 countries (Australia, France, Germany, The Netherlands, Norway, Spain, Sweden, Switzerland, UK and USA). The agencies that produced the highest number of reports, eight reports, was the Agency for Health Research and Quality from the US administration and the National Institute for Health and Research (NIHR) Health Technology Assessment (HTA) Program from the UK with also eight published reports. Regarding the country with highest number of reports was the UK with 10 reports. According to World Banks'^2^ classification all the countries with retrieved reports were classified as High Income Countries and all of them were OECD members (http://data.worldbank.org/about/country-and-lending-groups#High_income). Regarding the date of publication the period covered was 1994–2014. Thirty two reports out of 42 (76.2%) were published in the last 10 years, showing a trend to increase the number of publications around this topic. The highest number of publications was obtained in 2006 and 2009, the mean in the whole period covered was 2.1 and the mode 2.

**Table 1:** **Characteristics of considered reports**.

**Agency**	**Title**	**Year**	**Outputs**	**Economic analysis**
CADTH	Nutritional supplementation for patients with cancer: a review of the clinical effectiveness and guidelines	2014	Effectiveness	–
CADTH	Oral nutrition intake for the prevention of falls in older adults: clinical effectiveness and guidelines	2014	Effectiveness	–
SBU	Dietary treatment of obesity. A systematic review	2013	Benefits, risks, ethical considerations and Cost effectiveness	Cost effectiveness. Societal perspective
AHRQ	Interventions for feeding and nutrition in cerebral palsy	2013	Effectiveness	–
CEP	Nutrition during hospitalization for pediatric bronchiolitis	2013	Effectiveness	–
NIHR	Elemental nutrition for Crohn's disease	2013	Effectiveness and cost-effectiveness GRADE method for recommendations	Cost-minimization analysis and cost-effectiveness analysis.
NIHR	Adapting health promotion interventions to meet the needs of ethnic minority groups: mixed methods evidence synthesis	2012	Effectiveness	–
NOKC	The effectiveness of health promotion and preventive interventions on nutrition, physical activity, obesity and sexual health in children and adolescents	2012	Effectiveness	–
NIHR	The effectiveness of interventions to treat severe acute malnutrition in young children: a systematic review	2012	Effectiveness	–
GR	Guidelines for a healthy diet: the ecological perspective	2011	Sustainability	–
NIHR	CALORIES: A phase III, open, multicenter, randomized controlled trial comparing the clinical and cost-effectiveness of early nutritional support in critically ill patients via the parenteral vs. the enteral route	2011	Effectiveness and cost-effectiveness	Cost-effectiveness analysis from health and personal health services perspectives. Scale generic EQ5D
AHRQ	Nutritional research series: advancing the role of evidence-based reviews in nutrition research and applications Volume 2: issues and challenges in conducting systematic reviews to support development of nutrient reference values: workshop summary	2009	Methodology	–
AETMIS	Protein sparing modified fast diet: efficacy, safety and clinical use	2010	Efficacy, safety and clinical use	–
CADTH	Indirect calorimetry to measure energy requirements: a review of the guidelines and clinical effectiveness	2009	Clinical effectiveness	–
CADTH	n-3 lipids for patients on total parenteral nutrition: a review of the clinical and cost-effectiveness	2009	Clinical effectiveness and cost-effectiveness	Health care provider perspective, cost effectiveness
AHRQ	Nutritional research series: advancing the role of evidence-based reviews in nutrition research and applications. Volume 1: application of systematic review methodology to the field of nutrition	2009	Methodology	–
AHRQ	Nutritional research series: advancing the role of evidence-based reviews in nutrition research and applications. Volume 3: reporting of systematic reviews of micronutrients and health: a critical appraisal	2009	Methodology	–
IQWIG	Systematic guideline search and appraisal for the DMP module “Obesity”	2009	Methodology	–
GR	Healthy nutrition: a closer look at logos	2008	Effectiveness	–
AHRQ	Effectiveness and safety of vitamin D in relation to bone health	2007	Safety and effectiveness	–
AHRQ	Advancing the role of evidence-based reviews in nutrition research and science-based applications	2007	Methodology	–
NIHR	FOOD: a multicenter randomized trial evaluating feeding policies in patients admitted to hospital with a recent stroke	2006	Effectiveness	–
ZoNW	Cost-effectiveness of nutritional screening and intervention in elderly subjects after hip fracture	2006	Cost-effectiveness	Cost-effectiveness, societal perspective using a time horizon of 6 months. Scale generic SF36
Osteba	Development of an early detection and intervention system to prevent hospital undernourishment	2006	Effectiveness, prevention	–
GR	Guidelines for a healthy diet 2006	2006	Effectiveness	–
NICE	Nutrition support in adults: oral nutrition support, enteral tube feeding and parenteral nutrition	2006	Effectiveness	–
CEDIT	Nutrition support teams—systematic review, expert panel	2006	Effectiveness	–
GR	Food and nutrition in babies and young children. Review, expert panel	2006	Effectiveness	–
CEDIT	Nutrition support teams—preliminary report	2005	Effectiveness	–
ZONW	Activity, Lifestyle, and Nutrition and Therapy study (ALANT study)	2005	Effectiveness	–
AHRQ	Effects of omega-3 fatty acids on cardiovascular disease	2004	Effectiveness	–
CCE	Intravenous or nasogastric rapid rehydration for children with gastroenteritis?	2004	Effectiveness and safety	–
CCE	Assessing the effectiveness of total parenteral nutrition for simultaneous renal and pancreatic transplant patients	2003	Effectiveness	–
SBU	Geriatric care and treatment: a systematic compilation of existing scientific literature	2003	Effectiveness	–
AHRQ	Counseling to promote a healthy diet	2002	Effectiveness	–
CCE	“Open” vs. “closed” systems for enteral feeding	2002	Effectiveness and safety	–
CCE	Time to commencement of oral feeding following laryngectomy	2001	Effectiveness	–
ANAES	Care and monitoring of enteral access for enteral nutrition in adults in hospital and at home	2000	Effectiveness	–
TA-SWISS	Functional food	2000	Effectiveness	–
NIHR	Health promoting schools and health promotion in schools: two systematic reviews	1999	Effectiveness	–
NIHR	Home parenteral nutrition: a systematic review	1997	Effectiveness and cost-effectiveness	Health care perspective. Cost minimization and cost-utility
ANAES	Peri-surgical nutritional support (TPN or EN) in adults—consensus conference	1994	Effectiveness	–

Once the reports were analyzed we encountered as main characteristics that: 6 reports included analysis of cost-effectiveness, 6 reports methodological approaches, 6 around healthy diet, 1 functional food and 1 publicity analysis.

### Economic studies

NIHR (Richards et al., 1997). This study compared home parenteral nutrition (HPN) and in-patient care, the main conclusions of the study in the economic side were related to cost minimization as it was stated by the authors the HPN treatment was cheaper than the alternative of in-patient care. The perspective use was health care system. Regarding the cost per QALY, two studies retrieved by this review showed that that the cost utility of treating younger patients was more favorable than older ones.

ZonMW (2006) on cost-effectiveness of nutritional screening and intervention in elderly subjects after hip fracture, performed an economic evaluation from societal perspective using a time horizon of 6 months the scale to calculate the QALY was the generic SF36 (Ware and Sherbourne, 1992). For the experimental intervention of the study, the authors performed a detailed micro-costing study that was performed using the activity-based costing method. According to authors the incremental cost-effectiveness ratios were determined through: (a) direct costs of the intervention (food supplement, consultation by dietician) per day reduction in total length-of-stay; (b) total societal costs (including medical costs, patient costs, and caregiver burden) per QALY. *Post-hoc* subgroup analyses were performed to study heterogeneity and related subgroup cost-effectiveness. The results of the study were finally published in 2013 (Wyers et al., 2013).

CADTH (2009) report on n-3 lipids for patients on total parenteral nutrition, is a review of the clinical and cost-effectiveness, the authors used the health care provider perspective due to the retrieved articles. They included a cost-benefit analysis and impact budget analysis. The limitations of the review were related to the scarce number of economic studies that addressed the theme.

NIHR (2011) (CALORIES). It is a pragmatic, open, multicenter, randomized controlled trial comparing the clinical and cost-effectiveness of early nutritional support in critically ill patients via the parenteral vs. the enteral route. It is one of the first approaches to perform an economic analysis of different nutritional strategies from data directly obtained from a randomized trials and micro-costing. The cost analysis will take a health and personal services perspective as per guidance from NICE (2004, 2007, 2013). The results of this trial have been partially reported in different articles (Harvey et al., 2014a,b) and they are planned to be finished by the end of 2015 (Available in: http://www.nets.nihr.ac.uk/projects/hta/075203 accessed on January 2015).

Tsertsvadze et al. (2013) is a non-finished commissioned report on the “Elemental nutrition for Crohn's disease.” The systematic review includes an analysis of the effectiveness and cost-effectiveness and the authors plan to use GRADE method for the elaboration of recommendations. In the case of the economic analysis their approach will be cost-minimization analysis and cost-effectiveness analysis. The protocol can be consulted in: http://www.nets.nihr.ac.uk/__data/assets/pdf_file/0019/81820/PRO-13-08-01.pdf and the results are supposed to be published by mid 2015.

SBU (2013)^3^. This Swedish study around obesity and strategies to address the problem, performed by the Swedish Federal Office for HTA, tried to include all the domains of a HTA analysis. In the overview performed of health economic studies within the scope of this report, the authors indicated that there is a lack of well-executed studies relating to the cost-effectiveness of various types of dietary advice.

### Methodological studies

Maglione et al. (2007), Lichtenstein et al. (2009), Chung et al. (2009), Russell et al. (2009); see Table 1. The Agency for Healthcare Research and Quality's (AHRQ) is a governmental agency of the US that aims to produce evidence to make health care safer, of higher quality, more accessible, equitable, and affordable, and to work within the U.S. Department of Health and Human Services and with other partners to make sure that the evidence is understood and used. They produced a series of reports in order to guide any approach to the evaluation of nutrition research and its outcomes (Lichtenstein et al., 2008, 2009; Chung et al., 2009; Russell et al., 2009). They can be retrieved in: http://www.ahrq.gov/research/findings/evidence-based-reports/tr17-series.html They concluded that: (a) the methodological approach of “systematic reviews” is applicable to nutrition field; (b) there are problems of generalizability of well conducted studies to the overall population and appropriate interpretation and integration of scientific evidence from observational studies; (c) the reporting quality of SRs has improved 3 years after publication of SR reporting standards (since 2003), but the reporting of nutrition variables has not. Improved adherence to consensus methods and reporting standards should improve the utility of nutrition SRs.

IQWIG (2009). IQWIG is an organization commissioned by the German Federal Government to provide information on different topics for the German Healthcare System. They performed an analysis of the feasibility of the search on Clinical Practice Guidelines to inform and give recommendations regarding the role of nutrition and nutrition based interventions in obesity. They found that from the retrieved CPGs, RCT-based recommendations exist particularly in the care areas of nutrition therapy, physical activity therapy, behavioral therapy, pharmacotherapy and surgical therapy. In contrast, hardly any RCT-based recommendations could be identified for the care aspects of diagnosis, monitoring and long-term weight maintenance, or for care coordination and quality indicators.

## Discussion

### Study limitations

This is an overview of studies on nutrition and its consequences on health and wealth from the perspective of HTA and thus, it can be considered as a partial study of what is going on around nutrition and its possible impact on health and social care systems. In fact, we haven't considered the inclusion of broad databases such as Medline or EMBASE when looking for studies that address the effectiveness and cost-effectiveness of nutrition interventions. Nevertheless, searching the CRD is a recognized source when retrieving HTA reports or studies on effectiveness and cost-effectiveness (Royle and Waugh, 2003) from HTA doers and main agencies include their reports or articles in the three main databases comprised under the CRD interface (DARE, INAHTA, and NHS EED), but those produced by the British NIHR that are indexed in both the CRD and Medline (they appear under the name of Health Technology Assessment Journal). So any search that would have included Medline would have obtained duplicates for NIHR and no retrieves for other agencies but partial articles referring individual domains of full HTA reports indexed in the CRD. Moreover, in order to include all the possible reports, even those that were not indexed in the CRD, the authors sought for reports in the individual webpages of INAHTA agencies (HTA doers). HTA agencies or organizations, such as NICE or NIHR, no members of INAHTA also index their reports in the CRD databases.

### Information retrieval for systematic reviews in nutrition field and its validity for decision making process

Most of the retrieved reports correspond to the analysis of the effectiveness of dietary interventions and their consequences from the perspective of clinical effectiveness, some of them concrete and focused on pathologies and one concrete clinical setting (see Table 1) and did not address a more broader approach related to overall consequences on health care and social systems. Nevertheless, those that have reported this broader perspective (IQWIG, 2009; SBU, 2013) found difficulties in covering domains that are usual in HTA analysis such as: legal, organizational, economic, ethical and social aspects. The reason was the lack of evidence (research studies) referred to these domains.

It is worth pointing out that the number of randomized studies published in the research field of dietary treatment and its consequences has grown exponentially since the mid-1990s (SBU, 2013). At least about a thousand randomized studies have been carried out by now, especially in the area of dietary treatment of obesity and diabetes type II. However, most of these have short follow-up times, small study populations, have not gauged compliance with the dietary advice given, or have been carried out in a manner which makes them difficult to interpret. Only a small number of studies compare the effects of two or more kinds of dietary advice with one another, and the majority of these have been published over the past decade. These findings are in accordance with what was previously described in another systematic review reported around the domain of health economics (Gyles et al., 2012).

Most striking as regards diet comparisons is the lack of outcomes important to patients, such as morbidity, death and quality of life. These issues are of key importance when performing studies that aim to address the validity of health interventions and the possibility of providing evidence to decision makers. Nonetheless, it is true that nutrition is an area of research in which there is a requirement of long-term follow-ups and well conducted studies that avoid biases in order to establish correlations to final outcomes and many studies included surrogate outcomes or endpoints (cholesterol, triglycerides, glucose amount or changes on Body Mass Index) as indicators of the effectiveness of the intervention (Lenoir-Wijnkoop et al., 2011).

Another concern related to the lack of studies that address the indirect consequences of nutrition interventions is the lack of quality of life measurements that include social and/or well-being statuses; in fact, there is a vivid debate on how to address this and well recognized public bodies like NICE and MRC are commissioning researches on this theme (Improving cross-sector comparisons: Beyond QALY, 2015)^4^. There are a number of proposed sector-specific tools available; in social care, the Adult Social Care Outcome Tool (ASCOT) has been developed for routine use in social services. In public health, there is no single measure, but there are a number of broader measures that could be used. These include measures of wellbeing such as the preference-weighted ICECAP capability index (Flynn et al., 2015), WEMWBS (Tennant et al., 2007) and the ONS-4 (Office for National Statistics, 2014). So, there is a need to define a broader instrument that allows the comparison among interventions on health or that affect health and well-being beyond the QALY concept and perhaps refine the currently applicable thresholds to many healthcare systems' interventions.

Finally, as it was described in the approach to methodology relevant to nutrition research reported by AHRQ, it is difficult to achieve an appropriate balance between well conducted studies and applicability at a broader population (external validity) and the integration of evidence obtained from well-conducted observational studies with long-term follow-up periods (internal validity). In this sense the implementation of new methods for systematic reviews elaboration that include clinical judgment such as GRADE (Guyatt et al., 2008) and its applicability to HTA (Ibargoyen-Roteta et al., 2010) and public health interventions (Weightman et al., 2005; Craig et al., 2011) could be helpful to improve the quality and the use of SRs regarding nutrition research and interventions.

### Conflict of interest statement

The authors declare that the research was conducted in the absence of any commercial or financial relationships that could be construed as a potential conflict of interest.

## Footnotes

^1^HTA glossary. Available online at: http://htaglossary.net/HomePage Accessed on January 2015.

^2^World Bank's classification of countries and lending according to GNI per capita. Available online at: http://data.worldbank.org/about/country-and-lending-groups#High_income. [Accessed on January 2015].

^3^SBU. (no 218/2013). Dietary treatment of obesity. A Systematic Review. Available online at: http://www.sbu.se/upload/Publikationer/Content1/1/Dietary_treatment_obesity.pdf

^4^Improving cross-sector comparisons: Beyond QALY. MRC (2015). Available online at: http://www.mrc.ac.uk/funding/how-we-fund-research/highlight-notices/improving-cross-sector-comparisons-beyond-qaly/ [Accessed on April 2015].
